# Chemical Mixtures: Greater-than-Additive Effects?

**DOI:** 10.1289/ehp.114-a517

**Published:** 2006-09

**Authors:** Gerald A. LeBlanc, Guirong Wang

**Affiliations:** Department of Environmental and Molecular Toxicology, North Carolina State University, Raleigh, North Carolina, E-mail: ga_leblanc@ncsu.edu

Various combinations of chemicals are being detected in the environment with increasing frequency. This has raised awareness that we are not exposed to individual chemicals in isolation and heightens concern that the toxicity of individual chemicals may not represent toxicity when the chemicals are present in combination. Of greatest concern is that chemicals in combination may elicit synergistic toxicity that goes undetected in evaluations of individual chemical toxicity.

In a recent article, Hayes et al. (2006) assessed the effects of nine pesticides individually (at 0.10 ppb) and in combination (each at 0.10 ppb) on time to foreleg emergence and time to complete tail resorption in *Rana pipiens*. Both end points are measures of larval development in frogs. The authors reported that the pesticide mixture had a much greater effect on these developmental parameters than did the individual chemicals; they concluded that estimating ecologic risk of pesticides on amphibians using studies that examine single pesticides may lead to gross underestimates of the role of pesticides in amphibian declines. Clearly, Hayes et al. implied that the combined effect of the nine pesticides is greater than the sum of the individual chemicals. But is this speculation of synergy warranted from these data?

To invoke synergy, one must—at a minimum—exclude the possibility of concentration or response additivity. Concentration additivity may appear as synergy when individual constituents, sharing the same mechanism of toxicity, in a mixture are all present below the threshold concentrations required for toxicity. However, in combination, the joint concentration of the constituents exceeds that threshold concentration, resulting in significant adversity. These experiments were not designed to assess concentration additivity, so no judgment can be made either in favor of or against the possibility that the toxicity of the mixture represented concentration additivity. However, individual responses to the nine pesticides were shown in graph form (Figure 1; Hayes et al. 2006), which allows for an assessment of response additivity for the mixture. Eight of nine pesticides prolonged the time to foreleg emergence, and nine of nine chemicals prolonged the time to tail resorption. However, these effects were not statistically significant, with the exception of the effects elicited by propiconzole.

We subjected these data to analyses for response additivity under the assumption that the observed effects were real but were not statistically significant due to the low power of the experimental design. A description of the response additivity model is available on our website [Computational Approach to the Toxicity Assessment of Mixtures (CATAM) 2006a] along with a mixtures toxicity calculator used in these analyses (CATAM 2006b). The response addition model predicted that the mixture of pesticides would prolong the time to foreleg emergence from 44 days to 60 days and the time to tail resorption from 56 days to 67 days. Hayes et al. (2006) determined that the pesticide mixture extended these developmental time points to 59 ± 2 days and 70 ± 2 days, respectively (mean ± SE; these values are our best estimates from Hayes et al.’s Figure 2). Thus, response addition alone explains the toxicity associated with a pesticide mixture. There is no need to invoke greater-than-additive effects and no need to raise concern that mixtures of these pesticides cause unexpected toxicity.

Toxicity of mixtures is a perplexing problem that warrants significant investigation. However, when assessing the toxicity of chemical mixtures, it is prudent to test the null hypothesis of no interactions. Only upon rejection of this hypothesis should the possibility of synergistic interactions be considered. In response to the question posed by Hayes et al. (2006) in the title of their article—”Pesticide Mixtures … Are We Underestimating the Impact?”—the evidence presented suggests that the answer is “no.”
